# Comparing Prion Proteins Across Species: Is Zebrafish a Useful Model?

**DOI:** 10.1007/s12035-024-04324-z

**Published:** 2024-06-25

**Authors:** Anna Burato, Giuseppe Legname

**Affiliations:** Laboratory of Prion Biology, Department of Neuroscience, Scuola Internazionale Superiore Di Studi Avanzati (SISSA), Trieste, Italy

**Keywords:** Prion protein, Animal models, *Danio Rerio*, PrP1, PrP2, PrP3

## Abstract

Despite the considerable body of research dedicated to the field of neurodegeneration, the gap in knowledge on the prion protein and its intricate involvement in brain diseases remains substantial. However, in the past decades, many steps forward have been taken toward a better understanding of the molecular mechanisms underlying both the physiological role of the prion protein and the misfolding event converting it into its pathological counterpart, the prion. This review aims to provide an overview of the main findings regarding this protein, highlighting the advantages of many different animal models that share a conserved amino acid sequence and/or structure with the human prion protein. A particular focus will be given to the species *Danio rerio*, a compelling research organism for the investigation of prion biology, thanks to its conserved orthologs, ease of genetic manipulation, and cost-effectiveness of high-throughput experimentation. We will explore its potential in filling some of the gaps on physiological and pathological aspects of the prion protein, with the aim of directing the future development of therapeutic interventions.

## Introduction

The human *PRNP* gene, located on chromosome 20, encodes a 253 amino acid precursor protein containing two exons, with the entire coding region being delimited in the second one, thus excluding possible alternative splicing [1]. The mature cellular prion protein (PrP^C^) is formed by the removal of N-terminal and C-terminal signal peptides (amino acids 1–22 and 232–253), and by the addition of a glycosylphosphatidylinositol (GPI)-anchor at the C-terminus, which attaches the protein to the outer leaflet of the plasma membrane [2–4].

The structure of PrP^C^ is now well characterized, showing a N-terminal domain, consisting of an octarepeat region and a hydrophobic section, and a C-terminal domain, containing three α-helices (α1-3), three short segments of ß-strands (ß0 [5], ß1, and ß2), a disulfide bridge between amino acids 179 and 214 which comprises two N-linked glycosylation sites at the amino acids 181 and 197 [2, 6–9].

The cellular prion protein is an ubiquitously expressed protein mainly found in brain tissue, largely in the gray matter, but also in non-neuronal cells such as choroid plexus cells, ependymal cells, and endothelial cells of brain vessels [2, 6, 10]. Moreover, its expression has been reported in astrocytes [11–15], oligodendrocytes [15, 16], and microglia [10], in cells of the immune system like lymphocytes and mast cells [17, 18], and in many other body compartments such as heart, liver, intestine, and kidney [6, 12, 16, 18].

This widely spread expression suggests a plethora of physiological functions in which PrP may be involved [6], among which neuritogenesis [19, 20], cell signaling [21, 22], cell adhesion [19, 23], response against stress [24–26], circadian rhythms [27], recovery from sleep deprivation [28], neural stem differentiation in the central nervous system [15, 29, 30], myelination in the peripheral nervous system [31], and synaptic plasticity [32].

However, this protein aroused interest among researchers mainly for its role in neuropathology. In fact, the cellular prion protein (PrP^C^) can structurally convert into a misfolded isoform, commonly termed PrP *Scrapie* (PrP^Sc^), through a posttranslational process during which it acquires a higher β-sheet content [8, 33–35]. This proteinaceous infectious particle devoid of nucleic acid, formed largely, if not entirely, by the cumulation of PrP^Sc^, is defined as *prion* [36], characterized by transmissibility among individuals from the same and often different species and resistance to proteinases, heat, or conventional decontamination methods that modify nuclei acids (distinguishing it from viruses, plasmids, and viroids) [37]. Aggregation of prions in the central nervous system is the molecular hallmark of *prion diseases*, a class of rapidly progressive and transmissible neurodegenerative disorders affecting humans and other mammal species [2, 33, 38]. These diseases, which are also called transmissible spongiform encephalopathies (TSEs), include among others Creutzfeldt–Jakob disease (CJD), kuru, Gerstmann–Sträussler–Scheinker syndrome (GSS), and fatal familial insomnia (FFI) in humans, and scrapie, bovine spongiform encephalopathy (BSE), and chronic wasting disease (CWD) in animals [39].

Both in humans and animals, this misfolded protein can also act as a template, recruiting other cellular PrP molecules and converting them into their pathological counter forms in a deleterious cascade of events, enabling the disease to spread [40]. Moreover, many neurodegenerative diseases, such as Alzheimer, Parkinson, and frontotemporal dementia, show a similar prion-like protein misfolding and aggregation mechanisms [41]. In addition, the cellular prion protein seems to act as a toxicity-transducing receptor for the fibrillar aggregates involved in these diseases [42]. Specifically, in neuronal cells, PrP^C^ can mediate the toxic effects exerted by prions [43, 44], β-amyloid [45, 46], tau [47–49], α-synuclein [50, 51], and TDP-43 [52].

The rapid intercourse of prion diseases, as well as the involvement in other neurodegenerative diseases, opened a new series of questions concerning this protein.

In the majority of human cases, prion diseases arise sporadically with the spontaneous conversion of PrP^C^ into PrP^Sc^ [38, 53–55], while a smaller percentage is inherited, associated with mutations in the open reading frame of *PRNP* [39, 56]. In both cases, solving the 3-dimensional structure of PrP is crucial to discover the biochemical mechanisms leading to their misfolding.

Few data are available on the 3D structure of PrP^Sc^, since X-ray crystallography or NMR spectroscopy have to face the problem of the insolubility of the scrapie form, besides the heterogeneity of the samples [57, 58]. However, β-sheet enrichment, as a result of the PrP^C^-PrP^Sc^ conversion process, has been demonstrated by optical spectroscopy and structural prediction methods through the combination of computational analysis and available biochemical and genetic data [59, 60]. The beta structures, arranged to form left-handed ß-helices [61], have been also characterized with low-resolution techniques [62] and secondarily proven with the analysis of seeded fibrils exploited to label structures in brain tissue [63, 64]. Cryo-EM measurements showed that PrP^Sc^ may have a four-rung ß-solenoid architecture arrangement [65], which could explain the high content of ß-sheets and the resistance to protease digestion [58], specificities that differentiate it from the physiological counterpart. However, this structure has been doubted [66], highlighting the unresolved issues still ongoing on the matter. Recently, Cryo-EM high-resolution structures of mammalian prions have become increasingly more available [67–69], providing important clues on the structural characteristics that may account for their pathogenicity [70]. Despite paving the way for interesting future directions, significant gaps in understanding the molecular characterization of different strains, as well as the replication mechanisms and the species transmissibility barrier, still persist.

On the contrary, taking advantage of different animal species, in which PrP^C^ structure is conserved, a consistent amount of structural data is available for the cellular isoform [1, 3, 7, 8, 34, 35, 71–73], on which studies have been strongly focusing to better understand the biochemical mechanisms leading to the misfolding event.

## Conserved PrP Structure Among Different Animals

The primary structure of PrP^C^ is highly conserved among different mammals [34, 71, 72, 74], allowing the usage of different animal models to get new insights into the molecular mechanisms leading to the pathology.

Bacterially expressed recombinant PrP (recPrP), despite lacking Asn-linked glycosylation at residues 181 and 197 [75], is structurally equivalent to PrP^C^, and it has been used to gain more structural insights into the 3D structure by NMR and X-ray crystallography. The full-length form of PrP has a peculiar structure, conserved among different species: the N-terminal domain (amino acids 23–127) is intrinsically disordered and unstructured, while the C-terminus (amino acids 128–231) is folded into a globular conformation, with predominantly alpha-helical conformations and little ß sheet content [8, 73, 76].

As described above, the N-terminal is characterized by an octapeptide region, with a distinctive consensus sequence (PHGGGWGQ), which is a determinant in metal binding through the histidine residues, and it is involved in stabilizing the full-length protein, preserving its native folding [71, 77–79]. In addition, the hydrophobic section, in a region denoted as the non-octarepeat (non-OR) region, was evaluated for the association between histidine residues and copper binding in the conversion propensity of PrP^C^ to PrP^Sc^, with controversial results [7, 9, 71, 79–81].

The first animal PrP structures characterized with NMR were mouse PrP (MoPrP) [82] and hamster PrP (ShPrP) [83], both showing a high 3D similarity with human PrP (HuPrP) [8]. Both full-length proteins have overlapping structural domains with the human 3D assembly, along with the metal binding regions discussed above, a fundamental similarity allowing animal studies to be reliable also for human purposes.

The NMR structures of other mammals have been resolved, such as cats, dogs, pigs, and the two polymorphisms in sheep, which all share a specific conserved architecture of the globular domain with some local structural variations accounting for the different disease susceptibilities [84]. The rabbit, which instead is proven not to be susceptible to prion diseases [85], has some specific characteristics, such as the unique distribution of surface electrostatic potential, that could account for the missed conversion [86]. This last specific example further explains why structural studies are of fundamental importance for understanding the molecular mechanism of pathological conversion.

The species barrier in prion diseases, a complex and multifaceted mechanism, can be influenced by various factors, including the degree of homology of the prion proteins between the recipient and host species [72, 87], prompting the need for an analysis on the amino acid level.

The evolutionary conservation of the prion protein was addressed, finding a high level of amino acid sequence identity within mammals and birds: in particular, the high degree of conservation of the flexible N-terminal domain highly suggests its biological relevance [72].

**Fig. 1:**
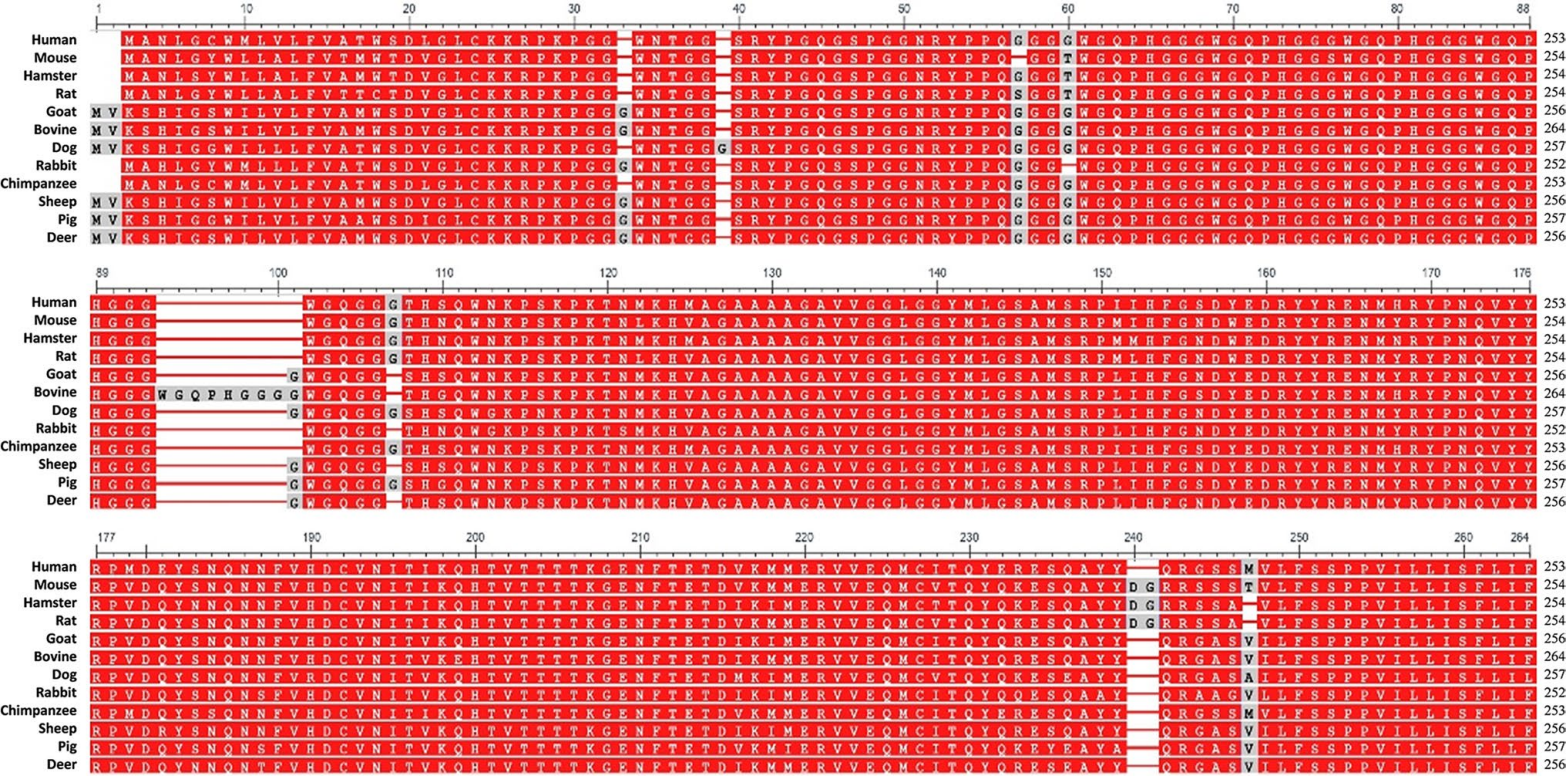
Multiple alignment showing the high similarity between PrP sequences among mammals. Red corresponds to highly conserved columns, while blue corresponds to less conserved ones (see the “Methods” section for program reference)

The amino acid sequence of full-length PrP is almost identical among mammals (Fig. 1).

The 3D structure similarity and almost identical amino acid sequence, besides their natural availability and easy maintenance, led rodent models, and mainly the mouse one, to be the most utilized throughout the years.

In particular, a great advantage was brought by transgenic PrP knockout mice generated with homologous recombination in embryonic stem cells, by disrupting the open reading frame, such as the Zürich I or Edinburgh lines, or by an extensive deletion of the gene, such as the Zürich II or Nagasaki [37, 88]. The heterogenous genetic background of the different lines, along with the different phenotypic effects due to probable off-target effects, hampered the physiological research on the protein [89]. To solve this problem, a Zürich III line was generated with the TALEN-genome-based technology, allowing a line that lacks the genetic confounders and artifactual phenotypes of non–co-isogenic *Prnp*^−/−^ lines [88]. The development of new knockout models, together with recent genomics developments, ushered the way to new experimental tests which started to bridge some of the gaps that this raging disease had opened. Specifically, genome editing techniques allowed a better understanding of the physiological role of PrP^C^ [22, 28, 31, 90–93], more insights on the specific regions of the protein that could be involved in the prion conversion [94–101], and addressed some questions regarding the species barrier in terms of transmissibility [102–107].

## Beyond the Mammalian Prion Proteins: Zebrafish

Our knowledge, in parallel, has been expanding toward the use of other animal models, not only among mammals, but also birds, reptiles, amphibs, and fish. In fact, species’ evolutionary distance from humans also showed the conserved 3D structure of PrP obtained from NMR studies. For example, chickens, turtles, and frogs clearly show extensive similarities with the 3D structure of human PrP, despite the lower amino acid matching profile [108]. More recently, *Danio rerio* has attracted attention among researchers, showing different homologs of the cellular prion protein, all with their own characteristics and similarities.

*Danio rerio*, commonly known as zebrafish, is a tropical omnivorous freshwater fish originating in northern India. This species has acquired a great biomedical importance, as shown by its increased usage as a research model throughout the years [109]. There are several reasons which make the zebrafish a great model for mirroring the development, in health or disease, of species of interest for humans. For example, a great advantage is given by the rapid *ex utero* development, as well as the low cost and extremely small size. The range of few millimeters to 3–5 cm in length, together with the high reproduction rate, allows large-scale genomic studies, since a great amount of animals can be housed in the same space [110]. Moreover, the optically transparent embryos, genetically tractable [111], permit the real-time imaging of the various developmental stages, allowing to visualize phenotypes in vivo at single-cell resolution [112]. Furthermore, their high tolerance to DMSO allows drug discovery studies very early in the disease process, assessing, besides their effective functioning, also their possible off-target effects [113, 114].

So, even if this model was perfectly fit for developmental studies, it is nowadays largely used also to get more insights into many of the human diseases [114–117].

Gene maps showed that there are many blocks of conserved syntenies between the two species, and zebrafish chromosomes (similar also in number) are orthologous to many human chromosomes. However, gene orders were often inverted and transposed, meaning that chromosome inversions have frequently been fixed in diverging populations in the lineage leading to zebrafish [118]. Moreover, the zebrafish reference genome sequence compared to the human genome showed that 70% of human genes have at least one zebrafish ortholog [119].

As the purpose of the review is addressing the potential of the model in studying prion diseases, it is important to specify that the brain has been classified as one of the zebrafish organs more similar to the human one [120, 121], recognizing that it can be exploited for modeling human neurodegenerative disorders.

With these premises, the solved neural development pattern, a well-characterized neuroanatomy, and the fully sequenced genome were a unique starting point for the investigation of the human prion-related pathobiology: although no prion replication has been found in this animal model to date, mutant strains and transgenic fish lines have become primary research tools to investigate the implications of specific genes or molecular pathways in the resembled mammalian pathology.

Zebrafish has two main PrP orthologs, defined as PrP1 and PrP2, which are twice the length of the mammalian protein but resemble many features of PrP structure [122].

Downstream of each fish PrP loci identified duplicated genes encoding short GPI-anchored polypeptides, suggested as PrP-like genes, denoted as PrP-rel-1 and PrP-rel-2 [122–124]. The latter, called PrP3 [125], has been investigated due to its structural peculiarities, which will be further discussed here, while PrP-rel-1 will be set aside for the purpose of this review.

**Fig. 2:**
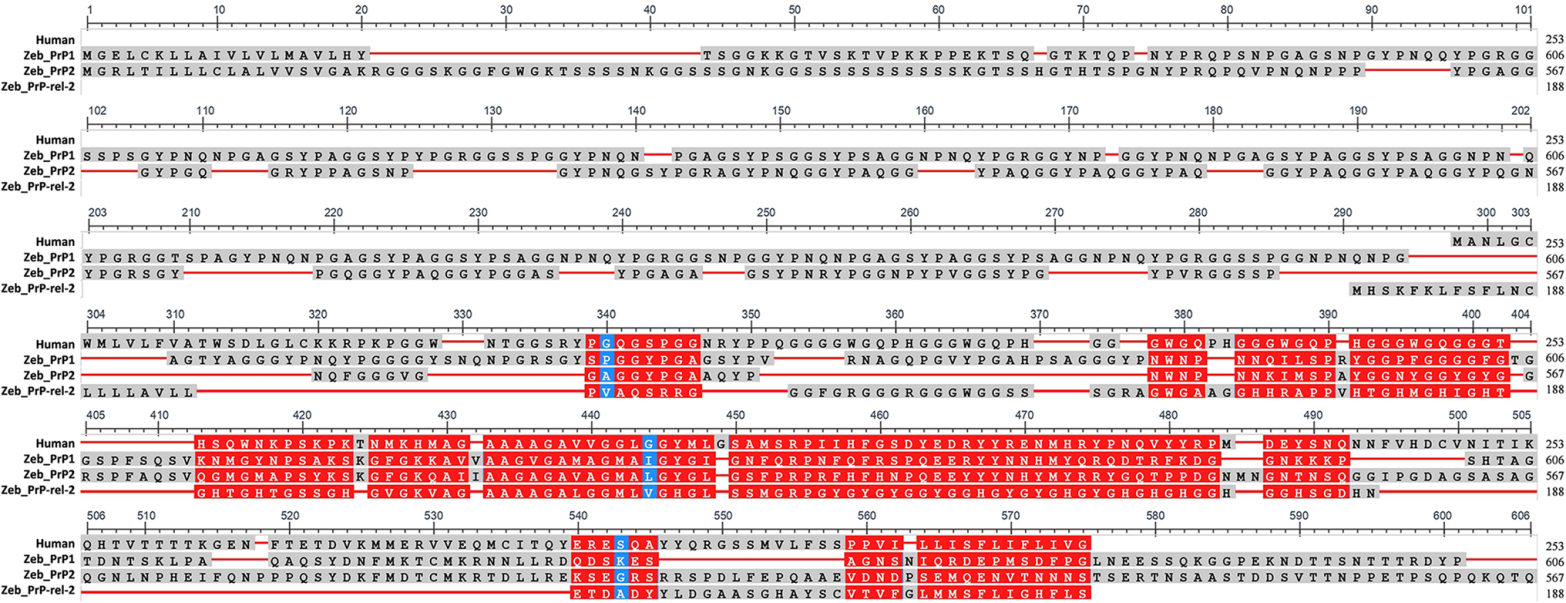
Comparison between human and zebrafish full-length PrP homologs. Red corresponds to highly conserved columns, while blue corresponds to less conserved ones (see the “Methods” section for program reference)

To first assess the importance of the zebrafish model to study the physiological function of PrP and its involvement in prion pathology, it is crucial to understand how consistent the homology with the mammalian protein is, to justify the parallelism (the sequences are compared in Fig. 2).

Both PrP1 and PrP2 show the highest similarity in the C-terminal domain, with the latter being slightly more analogous to human PrP (HuPrP) than the former. In the N-terminal domain, PrP1 has a repeat region (9 long repeats) similar to the octarepeat region of HuPrP, which is instead not found in PrP2. However, a Tyr-Pro-rich domain consisting in 18 hexapeptide repeats plus seven repeats with an irregular amino acid sequence length was identified in PrP2 as well. Still, none of them shows the presence of histidine residues in the repeat domain, further distancing the similarity with the possible conversion agents involved in the mammalian pathology [124]. Interestingly, the unique feature of both isoforms is the presence of a highly conserved 13 residue stretch between the repetitive region and the hydrophobic motif, which have neither predicted secondary structures nor similarity to known proteins [122].

PrP3 seems instead more distant, lacking some distinctive elements of mammalian PrP including the repeat domain before the hydrophobic central motif [124, 126]. However, a hexapeptide repeat domain was found after the hydrophobic motif, rich in histidine residues, highlighting a possible similarity with the human PrP, despite its different position [127, 128]. In detail, the structure was firstly predicted based on the NMR structure solved for HuPrP, and both PrP1 and PrP2, respectively 606 and 567 amino acids in length, show a signal peptide (residues 1–23/1–19, respectively), a long stretch of repeats (53–335/74–246), a hydrophobic central motif (379–395/299–315), two cysteine residues possibly implicated in the formation of the disulfide bond (residues 463 and 554/399 and 509), two asparagine residues that are significant putative N-glycosylation sites (residues 367 and 445/438 and 443), and a predicted hydrophobic C-terminal transmembrane region (residues 592–606/549–567). PrP3, instead, appears more evolutionary distant than the other variants, not only for the inversed position of the repeat domain and the hydrophobic region, but also for the absence of two of the three α helices and the second β strand [124]. However, it is included in the family of short GPI-anchored proteins, since it contains PrP features such as a highly conserved hydrophobic domain, a β-1 stretch, and degenerated repeats [122].

## Into the Physiology: PrP1 and PrP2

To assess the functions of PrP1 and PrP2, it is crucial to explore the developmental expression pattern of the two isoforms. This is particularly important due to the high similarity of the two aminoacidic sequences, possibly highlighting redundant functionalities. The different expressions in the nervous system could raise some questions on the evolutionary purposes of having two separate homologs, pointing toward unique and specific functions for each of them.

PrP1 transcripts were first detected with in situ hybridization in the floor plate [124], a specialized glial structure situated at the ventral-most part of the vertebrate neural tube that controls the regional differentiation of neurons in the nervous system [129], but this signal is lost after 3 days post-fertilization (dpf). From 48 h post-fertilization (hpf) to the larval stages, a strong signal is identified in cranial ganglia, including the trigeminal ganglia and their projections, while by 8 dpf, the cranial cavity shows PrP1 transcripts. Differently, a high level of PrP2 mRNA is detected in blastomeres and in the embryo from the mid-blastula transition to the end of the segmentation period [124], even if in the early mid-blastula stage, other authors identified the presence of PrP1 instead, leading to conflicting results [130]. Moreover, up to larval stages, and later in development, the expression pattern of PrP2 is widely distributed, detected in several distinct anatomical structures, and extensively spread not only in the nervous system but also in other body compartments [124, 130].

As commonly accepted, there is an important difference not only in the spatial distribution, but also in the temporal expression development of the two isoforms: in fact, PrP1 has a highly spatially restricted expression in the central and peripheral nervous systems in the very early stages of development, suggesting an implication in cell division and migration in the entire embryo, while PrP2 transcripts are found widely distributed within the CNS and in other anatomical structures more in the later stages, specifically upregulated in the developing nervous system [124, 130, 131].

Comparing the expression pattern of these two PrP orthologs provides relevant information on the relationship between evolutionary expression and functions, considering the structural similarities or differences among diverse PrPs [132]: the specific role of distinctive motifs could be addressed with models, such as the zebrafish.

To summarize, if the aminoacidic sequence and the defined structure point toward an overlapping between the functionalities of PrP1 and PrP2, the dissimilar spatiotemporal expression patterns, instead, lead the researchers to believe that these isoforms may not have the same evolutionary purpose.

To better investigate their unique roles, two KO models were generated. However, the main issue encountered with KO zebrafish models was the complete absence of the foreseen overt phenotypes, suggesting gene compensation mechanisms [126, 133]. Following this, morpholino (MO) antisense oligomers became the tool of choice for zebrafish gene knockdown (KD) [134], enabling the interference with PrP without altering their sequence [135], even if the potential of off-target effects should be further addressed [136].

In addressing the role of PrP1, different concentrations of MO were used in the early development of the embryos and, oppositely to KO studies which did not show any clear phenotype [126], the KD resulted in necrosis and developmental abnormalities, expressed mainly as alterations in CNS morphology [131, 137].

PrP2 KD, differently, mainly led to phenotypically defective midbrain and hindbrain development, with histochemical studies showing altered trigeminal ganglion morphology, reduced number of peripheral neurons and apoptotic cells with hyper-condensed nuclei [125]. In PrP2 KO studies, instead, the brain anatomy seemed conserved [133], thus further highlighting the probable gene compensation mechanisms underlying the development of the zebrafish devoid of the prion protein.

To additionally rule out the redundancy of zebrafish PrP functionalities, other selective KD of PrP1 and PrP2 and relative rescue experiments were performed. It was, for example, shown that PrP1 is selectively implicated in the gastrulation event, since the KD of this gene lethally arrested it, while PrP2 left the gastrulation process unchanged, while showing morphological defects [130]. This further highlighted the diverse implications of the two isoforms, seeing PrP1 more involved in the embryonic development, while PrP2 in neuronal differentiation and brain morphogenesis, coherent with the different expression pattern discussed above.

More in detail, the effect of PrP1 on the epiboly, a specific stage of the gastrulation process, was not only rescued by the addition of PrP1 transcripts, but partially also of both PrP2, still highlighting the similarity in the sequences, and surprisingly of mouse PrP [130]; this latter evidence showed that the zebrafish PrP and mouse PrP may not be that distant as theoretically suggested by the sequence comparison.

Since key factors that may play a role in gastrulation cell movements are the cell–cell adhesion processes [138], the implication of PrP1 was specifically addressed, showing how its absence caused important defects due to the progressive loss of E-cadherin from cell contacts [130, 131, 139]. Interestingly, selective depletion of the repetitive or the globular domains of PrP1 equally affected the epiboly, but the localization pattern was continuous when the former was deleted, while punctate when the latter was eliminated. The same punctate distribution was observed in PrP2 and mouse PrP globular domain KD: it may be concluded that the globular domain, as a conserved functionality among species, is crucial to ensure a continuous localization pattern. However, since WT PrP1 normally distributes in puncta, it can be also suggested that the long repeat domain of PrP1 has a stronger clustering activity than that of mouse PrP or PrP2 and that its globular domain is not strong enough to counteract the effect of the N-terminal motif [139].

Despite this difference with mouse PrP, PrP1 recalls the same cell-adhesion functions seen in mammalian studies, since, among the different PrP^C^ protein interactors, the neuronal cell adhesion molecule (NCAM) has been extensively characterized in vitro, in cell-based assays, and in vivo [140, 141]. Moreover, the role of PrP^C^ in E-cadherin-mediated cell–cell contact formation was confirmed in several human epithelial cell lines [142].

Taken together, these data suggest both similarities and dissimilarities between zebrafish and mammalian PrP, which need to be further investigated to better characterize the efficiency of these models, especially when addressing the pathology: however, PrP1 is so far proven to be certainly linked to the mammalian functions.

KO studies were able to clarify the non-redundant functions of the two zebrafish isoforms when investigating the posterior lateral line, a mechano-sensory system which exhibits a strong expression of PrP2 up to later developmental stages [124, 130]. In particular, the number of neuromasts was oppositely affected by the deletion of PrP1 or PrP2 genes, with the former deletion decreasing, while the latter increasing the number of these sensory organelles [126]. This reduction was also confirmed by knocking down PrP2, showing that this protein is necessary for the correct formation and stabilization of neuromasts in the migration process [143]. Since the number of neuromasts appears to be increased in transgenic zebrafish lacking β-secretase [144], the protease generating the mature β-amyloid from the ß-amyloid precursor protein, a possible link between zebrafish PrP and Alzheimer could be further addressed.

Moreover, seizure susceptibility, typical of diseases such as Alzheimer’s disorder, was strongly increased in PrP2 KO [126, 133], and interestingly, the deletion of both PrP1 and PrP2 genes decreased such effect, as if the simultaneous deletion of PrP1 was somehow hindering the outcome [126]. The role of PrP^C^ in modulating seizures and neuronal activity has been already seen in mouse models [145], suggesting a strong conserved function in neuronal maintenance and synaptic activity. As evidence of the implication of PrP2 in these functions, KO studies showed that transgenic fish exhibit learning deficiencies and age-dependent memory decline, implicating that PrP^C^ loss of function could be involved in disease-associated symptoms [146, 147]. Neural excitability mainly related to NMDA receptors was investigated in zebrafish prion proteins: PrP2 absence, both KO and KD, seems to disrupt the receptor dynamic [133], providing an explanation of the increased seizure susceptibility discussed above. Interestingly, the mammalian protein has been proved to also modulate these glutamatergic receptors, but through different molecular mechanisms: in fact, the mammalian protein is known to modulate NMDA receptors by binding copper via the repetitive region, exerting neuroprotection [148–153]. As already discussed, PrP2 has a partially equivalent repeat domain, but the lack of histidine residues resulted in the inability to bind copper, suggesting other molecular mechanisms exploited to exert the same mammalian function.

In summary, PrP2 is not only the most similar in sequence and structure, but it also parallels the mammalian PrP functions and exhibits very similar loss of function effects caused by its reduction. However, the molecular mechanisms underlying these functions may be distant between the species due to important protein structural dissimilarities, such as the presence or absence of histidine residues in the repeat domain.

In conclusion, the physiological functions of PrP^C^ have a great overlap in the zebrafish model, both in PrP1 and PrP2, but the link between the structural conversion and the pathological phenotype may be harder to address.

## Into the Pathology: PrP3

PrP3 could be possibly implicated in prion-related pathology, even if, as addressed previously, the aminoacidic sequence is distant to the mammalian counterpart, compared to the other homologs.

**Fig. 3:**
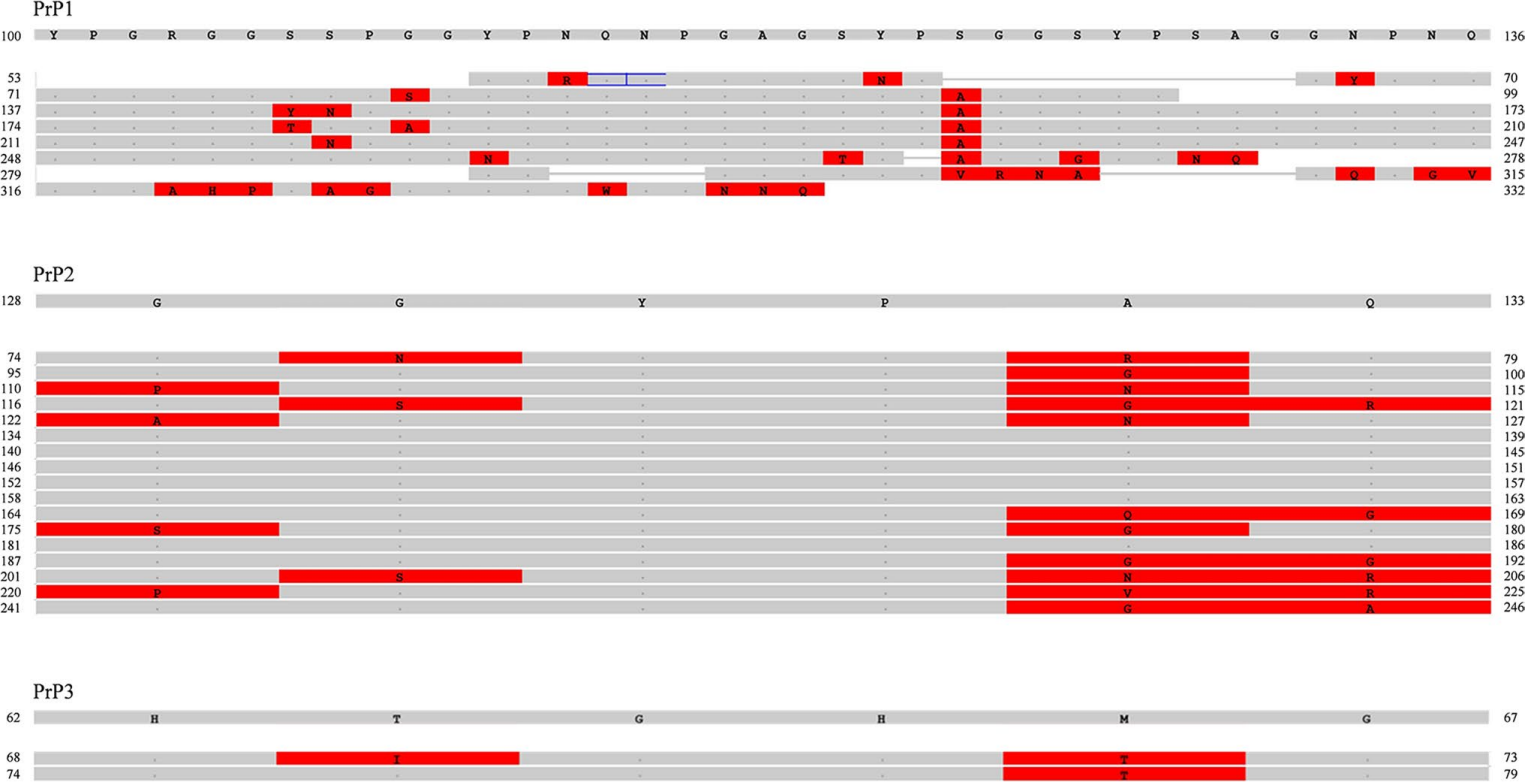
Zoom into the amino acids 53–335 of PrP1 to show the 9 long repeats, amino acids 74–246 of PrP2 to show the 18 hexapeptides, and amino acids 62–79 of PrP-rel-2 to show the 3 hexapeptides. The first line of each image represents the sequence anchor on which the repetitions are compared. Red shows mismatching amino acids, while gray is for identicalities (see the “Methods” section for program reference)

Interestingly, differently from PrP1 and PrP2, PrP3 showed the presence of histidine residues in the repeat domain (a comparison between repeat domains of the three isoforms is shown in Fig. 3). These histidines, which are numerous and in close vicinity, have been discovered to be potentially related to the pathological side of PrP, since they are able to bind copper and zinc as in the mammalian species [127, 128, 154]. The NMR structure of the fragment encompassing the copper binding site of this isoform has been solved, and the intra- and inter-repeat copper binding modes resulted to be even more effective than those of the mammalian octarepeat region [128]. Moreover, also the structural delineation of Zn^2+^ binding with the repeat domain of PrP3 was confirmed by NMR and molecular dynamics calculations [127]. The repeat domain in PrP3 is found after the hydrophobic region, an important structural difference to the mammalian protein that could lead to different pathological inferences [122, 124].

Although this disparity should distance the implication of this short protein in prion diseases, its relevance may still be addressed due to the PrP-like structural characteristics, such as a highly conserved hydrophobic domain, a β-1 stretch, and degenerated repeats [122, 124, 155].

Though, since it lacks some of the basic structural motifs, as already discussed, it was initially predicted to be an unstructured protein: however, their mammalian homologue Shadoo appeared to influence biological and pathogenic activities of PrP in vivo [156], further increasing potential importance.

PrP3 exhibits noticeable presence in embryonic cells before 24 h post-fertilization (hpf), but its expression diminishes in the developing brain as development progresses, being undetectable at 3 days post-fertilization (dpf). However, it is notably abundant in the central area of the pectoral fin buds, where PrP3 transcripts surpass those of PrP2. Additionally, PrP3 is found in the heart and branchial arches. Unlike PrP2, PrP3 is not found in significant levels in the central nervous system, kidney, liver, or posterior intestine during embryonic and larval stages. Consequently, the expression pattern of zebrafish PrP3 contrasts with that of tetrapod PrP, as it lacks prominent expression in the CNS [124].

Still, this PrP-like protein has not been consistently investigated so far, so many questions are yet to be answered.

## Conclusions: Is Zebrafish a Useful Research Model for Prion Diseases?

The primary pathophysiological characteristic of mammalian PrP involves the development of a misfolded, self-aggregating neurotoxic conformer.

Despite the absence of confirmed prion replication in zebrafish, the understanding of this phenomenon remains considerably limited compared to studies conducted in mammals. Additionally, the use of various mammal animal protein feeds in fish raises concerns about the potential contamination with mammalian prions, highlighting the need for cautious consideration [157].

To ascertain the suitability of zebrafish as a model for prion disease research, several factors need a thorough examination in light of the aforementioned evidence.

Firstly, while zebrafish PrP1 and PrP2 exhibit limited homology with their mammalian counterparts in terms of amino acid sequence, the 3D structural resemblance, along with their distribution patterns, offers an avenue for investigating the physiological functions of these proteins. This similarity may provide insights into the roles of these proteins, given the ease of managing zebrafish models.

The existence of two orthologs enables separate exploration of the molecular mechanisms underlying PrP1 functions, particularly during early gastrula, and those of PrP2, which are pertinent to developing neurons. This unique setup allows for a more nuanced understanding of the intricate interactions guiding specific events during development, a task that might be more challenging in mammalian models where a single protein must fulfill diverse functions across various stages.

Furthermore, the conserved 3D structure hints at the potential for zebrafish PrP to undergo conformational changes parallel to those seen in prion diseases, suggesting a novel path for studying prion pathology and its genetic underpinnings. This possibility could be further explored by efficiently inoculating transgenic zebrafish expressing mouse or human PrP with prions. Nevertheless, uncertainties persist regarding PrP3 and other prion-related proteins, which exhibit lower sequence homology and less conserved structures compared to mammalian PrP. Despite this, PrP3 retains important motifs observed in mammalian PrP, and specifically a repeat domain rich in histidine residues, which are central to prion disease studies. This further highlights the potential for zebrafish to acquire and possibly transmit prions, making it a potentially promising model for prion research, despite the absence of evidence regarding this capacity.

Regardless, while the inability to replicate prions may seem like a limitation, it does not necessarily preclude the model from shedding light on the pathological aspects of prion diseases. By assessing the similarities between the zebrafish and mammals in terms of protein structure, function, and biochemical pathways, researchers can explore the underlying mechanisms of prion pathology.

Of a great impact are the studies on protein–protein interactions, pathways, and exchanges with cell compartments, but also those on protein misfolding when undergoing conformational changes and the consequent neurotoxic effects induced.

The availability of a large number of zebrafish at a relatively low cost facilitates high-throughput genetic screens and sophisticated experiments, including real-time imaging of living animals, to elucidate physiological mechanisms relevant to prion pathology.

Furthermore, by unveiling the molecular and cellular processes involved in prion pathogenesis, researchers can identify novel targets for drug development and test the efficacy of therapeutic interventions. Additionally, these models can serve as platforms for screening candidate compounds and assessing their ability to modulate prion-related processes, ultimately leading to the development of effective treatments. Zebrafish’s tolerance to DMSO opens avenues for testing anti-prion therapeutic targets based on the specific molecular patterns identified.

In conclusion, the zebrafish model holds great promise: if it is primarily utilized thus far to unravel the physiological roles of PrP in the neuronal system, its potential extends to bridging the gap between physiological and pathological aspects of prion diseases, with the ultimate goal of informing therapeutic interventions.

## Methods

PubMed was extensively used for this manuscript, mainly with the advanced literature search (*protein name*) AND (species of interest).

UniProt was used to obtain all the amino acid sequences of the proteins mentioned in the manuscript, and COBALT: Multiple Alignment Tool was utilized for the comparison, as shown in figures. Links are as follows: https://www.uniprot.org/ and https://www.ncbi.nlm.nih.gov/tools/cobalt/re_cobalt.cgi.

## Data Availability

Not applicable.
